# In support of child and adolescent health research

**DOI:** 10.1038/s41467-022-32237-9

**Published:** 2022-08-10

## Abstract

To highlight and support research in the important area of child health, the editors at *Nature Communications* and *Communications Medicine* invite submissions to a collection of papers on this subject, including infectious diseases, cancer, and neurodevelopment and neurodiversity.

Historically, children have often not been the primary focus of medical and pharmaceutical research efforts, and in many cases research on adults has been repurposed for children. However, the way we think about childhood health and disease is changing. A shift in societal attitudes, funding priorities, legal and regulatory frameworks, as well as technological advances, are incentivising the research community to study childhood disorders as distinct entities and to enroll children into clinical trials for new pharmaceutical products likely to benefit them. For example, collecting data on the incidence and therapeutic outcomes of childhood cancer can contribute to advance our understanding of these diseases and have been exemplified by efforts such as the German Childhood Cancer Registry. Advances in genomic technology are being used to discover differences between adult and childhood cancers which in turn point to targeted therapies. Genomic sequencing has also revolutionized the diagnosis (and in some cases, treatment) of rare disease and developmental disorders, which often arise in childhood. Infectious disease spreading dynamics and epidemiology need to factor in the contribution of children and importantly, infections can result in different symptoms and syndromes when compared to adults. Optimization of dosing and treatment combinations of available drugs is important to adequately prevent and treat infectious diseases such as HIV and malaria in children. Finally, understanding that the immune system does mature and age has also dramatically changed our perspective on immune-related disease etiology and therapy.

Promoting the healthy development of children is crucial for the future of our societies. As an illustration of its importance, one of the targets within Sustainable Development Goal 3 in the United Nations 2030 Agenda for Sustainable Development is to end preventable deaths of newborns and children under 5. The global burden of child and youth deaths remains high, despite dramatic reductions in under-5s deaths, and there are stark inequalities across the globe in the availability of health services to children. In low and middle-income countries in particular, undernutrition is associated with a high percentage of deaths, and wasting and stunting remain serious concerns. Both malnutrition and obesity affect children in all countries across the world.

“The global burden of child and youth deaths remains high, despite dramatic reductions in under-5s deaths, and there are stark inequalities across the globe in the availability of health services to children.”

Moreover, the COVID-19 pandemic and the consequent disruptions to education and social interactions during key developmental stages have been linked to negative impact on mental health and wellbeing among children and adolescents. Understanding additional risk factors for mental health disorders, and investigating potential interventions to promote wellbeing and resilience in these age groups, are key to mitigating lasting effects of this experience.

The editors at *Nature Communications* and *Communications Medicine* wish to encourage submissions to their journals in the important area of child and adolescent health research. With our Open Collection, we want to highlight studies aiming to understand health and the transition to disease in children and adolescents, as well as to prevent and treat it once it occurs. Our interest in this topic is multidisciplinary, from basic research into the causes of childhood illness, to clinical diagnosis and therapies. Crucially, we also want to support research considering public health aspects including education and nutrition. We are looking forward to seeing how the research community efforts make progress in supporting child health.

